# The lingering impacts of COVID-19 on older women in Kenya and Uganda: A quantitative analysis of health, wellbeing, and WASH insecurity

**DOI:** 10.1177/22799036261474131

**Published:** 2026-08-07

**Authors:** Satveer Dhillon, Isaiah Omondi, Justine Nagawa, Elijah Bisung, Sarah Dickin, Kenneth Mugayehwenkyi, Diana Karanja, Susan J. Elliott

**Affiliations:** 1 Department of Geography and Environmental Management, Faculty of Environment, 124613 University of Waterloo, Waterloo, ON, Canada; 2 Reach One Touch One Ministries (ROTOM), Mukono, Uganda; 3 School of Kinesiology and Health Studies, 4257 Queens University, Kingston, ON, Canada; 4 Department of Women’s and Children’s Health, Centre for Health and Sustainability, 8097 Uppsala University, Uppsala, Sweden; 5 Community Health Support Programme, Kisumu, Kenya; 6 United Nations University Institute for Water, Environment and Health, Richmond Hill, Ontario, Canada

**Keywords:** COVID-19 pandemic, older women, Sub-Saharan Africa, WASH, health and wellbeing

## Abstract

**Background:**

The world appears to be experiencing more than its fair share of shocks: climatic disasters, pandemics, wars and political events that have far-reaching consequences for vulnerable populations globally. One such example was the COVID-19 pandemic, which resulted in both direct and indirect impacts on older women, especially those residing in Sub-Saharan Africa, due to the already fragile economic and health care systems in place, as well as the lack of infrastructure for public services. While substantial literature now exists documenting the impact of COVID-19 on vulnerable populations, less attention has been paid to building back better.

**Design and Methods:**

Using the feminist political ecology of health framework, this research investigated the impacts of the COVID-19 pandemic on health and wellbeing among older women residing in Kenya (n=231) and Uganda (n=211) and how they are ‘building back better’. Using survey data, we used a generalized linear model with a complementary loglog link function to identify factors associated with wellbeing, emotional distress and self-reported health status.

**Results:**

Only 17.7% of older women in Kenya and 14.7% in Uganda experienced an improvement in wellbeing post-COVID compared to during the pandemic. Further, there remains a sustained impact on emotional distress levels. Several factors, such as WASH insecurity and no health insurance, negatively impacted wellbeing, emotional distress and self-reported health status.

**Conclusion:**

Overall, this research documents how older women in Uganda and Kenya have been and continue to be impacted by the COVID-19 pandemic, despite declining case counts. Several policy recommendations have been outlined.

## Significance to public health

The findings of this work are critical to public health, as they address the urgent need to protect vulnerable populations against future global shocks. The COVID-19 pandemic exposed deep inequities, particularly for older women residing in Sub-Saharan Africa, who face compounded risks due to age, gender and lack of access to public infrastructure. By examining their experiences, this research informs policies and context-specific interventions that prioritize the voices and experiences of those most vulnerable. This research contributes to building better health systems and social safety nets that not only address current vulnerabilities but also mitigate the impact of future global health challenges.

## 1. Introduction

The frequency of unprecedented global shocks (financial, civil, geopolitical, environmental, climate related) has increased over the past few decades, affecting populations worldwide.^1^ One such shock was the COVID-19 pandemic, which had a profound and far-reaching impact on global systems and significantly affected the health and wellbeing of populations worldwide. With over 7 million deaths and more than 700 million people infected with the virus,^2^ the pandemic exposed systematic weaknesses in government preparedness, supply chains and public health systems.^3^ While the COVID-19 pandemic impacted countries across the globe, the impact was more pronounced in resource-limited settings, such as Sub-Saharan Africa (SSA), where systemic vulnerabilities were already present.^4^

Older adults in SSA represent the fastest-growing aging population in the world,^5^ yet they face intersecting challenges, including weak health infrastructure for older adults,^6^ inadequate WASH infrastructure,^7^ and limited social support systems.^8^ In SSA, older women continue to experience gendered ageism in accessing healthcare and community resources,^9^ while also facing disempowering conditions such as abuse, neglect, lack of political representation, and exclusion from civic life.^10^ All of these issues are further exacerbated by the limited financial support in these countries and the high demand for caregiving due to the HIV/AIDS epidemic,^11^ preventing older women from earning an income.^12^ During the COVID-19 pandemic, older adults faced compounding challenges, amplifying the health and wellbeing challenges faced by this vulnerable population. Notably, the WHO Regional Office for Africa (2021) reported that more than half of all COVID-19 deaths were those aged 60 and older.^13^ Hence, the *United Nations Research Roadmap for Building Back Better After COVID-19* declared that older adults, particularly older women, were experiencing the highest degree of marginalization as a result of the pandemic.^14^

Overall, older women in SSA bore a disproportionate burden during the COVID-19 pandemic, intensified by the pre-existing structural inequities. One major challenge arose when adult children working in urban centres migrated back to rural areas due to lockdowns, placing additional caregiving strain on older women.^15^ Furthermore, COVID-19 movement restrictions heightened the vulnerability of women’s livelihoods, especially among those relying on the informal sector, as many lost their jobs and sources of income.^16^ Even prior to the pandemic, older women faced challenges in meeting their own and their households’ Water, Sanitation, and Hygiene (WASH) needs, due to the physical demands of walking long distances and carrying heavy loads.^17^ These difficulties were exacerbated by COVID-19-related mobility restrictions, which increased WASH insecurity and negatively impacted older women’s quality of life.^18^ Additionally, their responsibilities for water collection placed them at greater risk of contracting COVID-19.^19^ Thus, the COVID-19 pandemic exposed and amplified structural inequities in health, the economy, and social protection, and intensified gender inequity in SSA, especially for older women.

Although numerous studies have examined the impact of the COVID-19 pandemic on older adults,^15,20,21^ far less is known about its older women in sub-Saharan Africa (SSA), who face distinct vulnerabilities including lack of access to basic services such as water, sanitation and hygiene, experience tremendous food insecurity and are also often raising the next generation of orphaned grandchildren due to the impacts of HIV/AIDS.^11,17^ In general, existing research highlights the unintended consequences of public health restrictions, particularly the adverse effects on older adults’ mental health due to social isolation during lockdowns (see Refs. 21 and 22). Studies have also highlighted the impact on healthcare utilization as transportation services were either closed to prevent the spread of the virus^15^ or were too costly.^23^ Despite these studies, there is a lack of focus on the sustained impact of the COVID-19 pandemic on older women’s health and wellbeing, particularly in SSA. 

This paper aims to address this gap by reporting the results of a quantitative investigation of the impact of the COVID-19 pandemic on the health and wellbeing of older women residing in Uganda and Kenya. The work is informed by the feminist political ecology of health framework that explores how gender and health are interconnected across various scales.^24^ It also examines how everyday practices and meanings contribute to patterns of gendered inequities, exclusion and marginalization.^25^

In this sense, this research explores how social and political factors exacerbate health and wellbeing outcomes among vulnerable older women residing in SSA. Specifically, there are three objectives: 1) to investigate the impacts of the COVID-19 pandemic on wellbeing, self-reported health status and emotional distress of older women in Uganda and Kenya; 2) to explore the determinants of those more likely to experience adverse effects on their health and wellbeing; 3) to examine access to WASH amenities as a determinant of health and wellbeing for older women in Uganda and Kenya. By addressing these three objectives, a roadmap can be developed to ensure vulnerable populations, in particular older women residing in SSA, are protected during future shocks.

The remainder of the paper is structured into five sections. After this introduction, we describe the research design and methods, including the selection of the study site and data collection techniques. The subsequent section presents the results, which align with the research objectives. The discussion then contextualizes the findings within the relevant literature, emphasizing the research, practical and theoretical contributions.

## 2. Research design and methods

### 2.1. Case study site

Located in East Africa, Uganda and Kenya have populations of 48.6 million and 55.3 million, respectively. In Uganda, the number of older persons in 2020 was 1.5 million, with older women representing 54% of this population.^26^ Ugandan older women are more likely to be single/widowed, reside in a skipped generation household, have lower levels of income, and have greater caregiving responsibilities compared to their male counterparts.^26^ In Kenya, the older persons population is 2.7 million, with women accounting for 55% of this rapidly growing group.^27^ Similar to older women residing in Uganda, older women in Kenya experience gendered ageism and lack access to work opportunities.^9^ The distinct and heightened vulnerabilities of older women in both countries underscore the importance of understanding how public health crises, such as the COVID-19 pandemic, have impacted this population.

Both Kenya and Uganda implemented various measures to combat the spread of the COVID-19 virus and mitigate its far-reaching consequences. In Uganda, the government enacted a swift and stringent lockdown in response to the first wave. This included restrictions on public transport, the closure of schools, and a nationwide curfew, all of which were aimed at curbing transmission.^28^ While these measures were effective in slowing the spread of the virus, they also had significant repercussions, disrupting various aspects of daily life and exacerbating existing socioeconomic disparities.^15^ In contrast to Uganda’s more restrictive approach, Kenya’s COVID-19 response has been characterized as more balanced, as the government sought to balance public health considerations with the need to maintain economic stability and livelihoods.^29^ For example, Kenya introduced movement restrictions only in counties that were considered epidemic hotspots.^30^

### 2.2. Data collection

The cross-sectional survey was conducted from May to August 2023. Funded by Canada’s International Development Research Centre, the Canadian Institutes of Health Research and the Social Sciences and Humanities Research Council's Women RISE initiative this project was conducted in partnership with Reach One Touch One Ministries (ROTOM) Uganda, COHESU Kenya and the University of Waterloo. ROTOM is a non-governmental organization (NGO) that supports the health and social care needs of older adults and their dependents in Uganda.^31^ COHESU is also a registered NGO that works to mobilize resources, provide training and conduct research to improve the health and wellbeing of individuals and communities in Kenya.^32^

**Figure 1.**
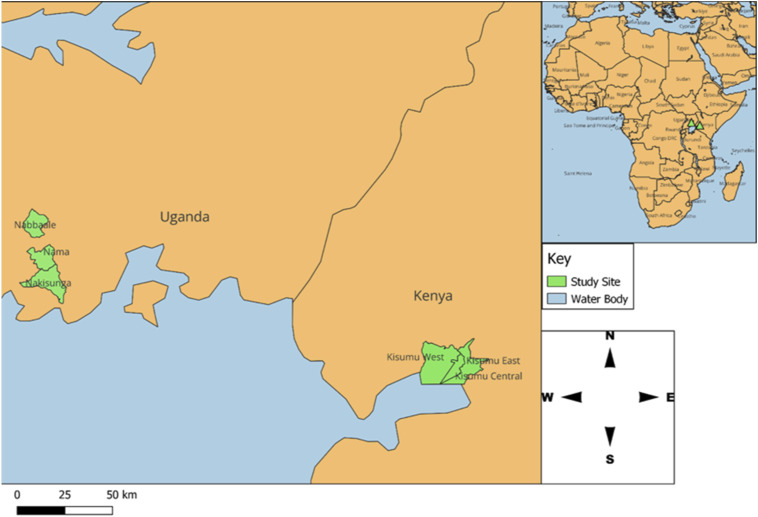
Study sites in Kenya; Kisumu Central, Kisumu East and Kisumu West sub counties and Uganda: Nabbaale, Nama and Nakisunga (Source: Isaiah Omondi (2023); generated using Q-GIS^1^ 3.28.3.

In Kenya, three rural and peri-urban sub-counties of Kisumu County on the shores of Lake Victoria were chosen as the research study sites: Kisumu East, Kisumu West, and Kisumu Central (Figure 1). In Uganda, three sub-counties of Mukono district were selected: Nama, Nakisunga, and Nabbaale (Figure 1). These communities were strategically chosen due to high levels of income inequality, with women being the most socioeconomically vulnerable.^33,34^

Participant recruitment followed a systematic process leveraging ROTOM’s and COHESU’ s established community networks. Surveys were administered by Community Health Promoters in Kenya and Village Health Teams (VHTs) in Uganda, using the KoBotoolbox platform, a form of Computer-Assisted Personal Interview software. All surveyors received appropriate training and piloting was conducted prior to implementation. No major issues were noted during this piloting, and hence no modifications were necessary prior to the commencement of the survey. On average, in Kenya, the length of a single survey was 20-40 minutes, and in Uganda, it was 30 minutes, with a range of 30-60 minutes. The data analyzed in this study was collected between June and August 2023 in both countries. Survey participants were given a token of thanks for their participation (a sugar cube and a bar of soap).

### 2.3. Sampling approach

Using purposive sampling, approximately 1000 women in each country were surveyed, representing a stratified (by age) sample with approximately 100 women in each age category: 16-20; 21-25; 26-30; 31-35; 36-40; 41-45; 46-50; 50-60; 60-70; 70+. The inclusion criteria were that the study participants had to be: 1) residents of the study site, 2) women, 3) 16 years of age or older, and 4) able to speak English, Swahili, or a local language. Although purposive sampling was used to identify women aged 16 years and above for inclusion, recording GPS coordinates helped ensure the participants were evenly distributed throughout the study area. Potential participants who met the above criteria and provided informed consent were included in the study. The sample size, n, for each age category/stratum was determined using Daniel’s formula: *n*=(Z_α_)^2^.p (1−p)/d^2^; where Z_α_ = 1.96, p = proportion of the population with access to clean water, taken arbitrarily to be 0.07, and d = error margin, taken to be 0.05. The resultant sample size was 100 per stratum and 1200 across the 10 strata, with a 20% oversampling margin to account for non-response in both countries. A total of 1135 and 1027 successful interviews were conducted in Kenya (response rate of 94.5%) and Uganda (response rate of 85.6%), respectively. For the purposes of this paper, all participants who were 60 years of age or older (n=442) were included. In Kenya, there were 231 participants: 124 in the 60-70 age range and 107 in the 70+ age range. In Uganda, there were 211 participants: 107 in the 60-70 age range and 104 in the 70+ age range.

### 2.4. Measures

The survey instrument consisted of ten parts, including basic demographic information, WASH access, empowerment in the WASH index, wellbeing, the Household Water Insecurity Access scale (HWISE), health status, and emotional distress.

Respondents were asked 12 questions related to WASH access (e.g. *what is the main source of drinking water for your household?*). We also included selected items from the Women’s Empowerment in WASH Index.^35^ These items include questions related to decision-making in WASH, group membership and access to capital. To support the analysis, a group membership index was created for both countries based on the responses obtained from the participants regarding their membership and participation to community groups (such as Water and Sanitation Users, Agricultural/livestock/fisheries producers, Credit/Microfinance, Religious, Educational, Trade and Business associations). Being a member of each group earned a score of ‘1’ plus another score of ‘1’ for contributing to decision-making. The scores were summed to generate an interval-scale variable for the analysis. Further, the wealth index was created based on the ownership of large and small consumer durable assets in households. Accordingly, sole ownership was assigned ‘1’, no ownership was assigned ‘0’, and the values were summed to generate the two interval wealth scale indices. The wealth scale demonstrated low internal consistency (Cronbach’s α = 0.63 in Kenya and 0.50 in Uganda) because only six items were captured during the survey and therefore included in the analysis.

Water and WASH insecurity scales were measured using the Household Water Insecurity Experiences Scale (HWISE) over the past 30 days. Water insecurity was measured using a set of eight questions, while WASH insecurity was measured using those eight questions plus four additional questions specific to sanitation and hygiene. Each question was scored on a five-point Likert scale of severity/frequency of occurrence: ‘never’, ‘rarely’, ‘sometimes’, ‘often’, ‘very often’. Numeric values were assigned to the responses as 0=Never/Rarely, 1=Sometimes, 2=Often, 3=Very often; the values corresponding to the respective questions for both scales were summed to generate two interval scales for the analyses. Both water insecurity and WASH insecurity scales had a Cronbach’s alpha value of 0.95 in Kenya and 0.94 in Uganda.

There were three outcomes: wellbeing, self-reported health status and emotional distress. Wellbeing was measured using a set of six questions scored in a five-point Likert scale (Never, Rarely, Sometimes, Often, Very often) reflecting the frequency with which the participants were unable to: *pay bills on time, pay health insurance premiums on time, pay rent on time, fix their houses, buy enough food, buy the thing they needed and the things they wanted, and not having enough money over the past year*. A numerical scale was generated by dichotomizing the options into 0 (Never/Rarely) and 1 (Sometimes/Often/Very Often), then summing them to create an interval-scale variable, with lower values indicating a better state of wellbeing. This study captured wellbeing in two instances – during the COVID-19 pandemic (captured retrospectively) and post-pandemic (reflecting the situation at the time of the survey). The change in these values was calculated and adopted as one of the outcome variables, with higher values indicating deteriorating wellbeing. Conversely, in instances where this value decreased, participants were regarded as having improved wellbeing and coded as ‘1’ for the regression analyses. The wellbeing scale had a Cronbach’s alpha of 0.81 in Kenya and 0.72 in Uganda during the pandemic, and 0.78 in Kenya and 0.73 in Uganda post-COVID-19 pandemic.

Self-reported health status was measured using the question “*rate the state of your health relative to others your age*” with possible responses being ‘excellent’, ‘very good’, ‘good’, ‘fair’ and ‘poor.’ These responses were further dichotomized into ‘Excellent/Very good/Good’ and ‘Fair/Poor’ for further analyses as one of the outcome variables.

Emotional distress was measured using the 12-item General Health Questionnaire (GHQ-12). The GHQ-12 scale includes a set of twelve questions on recent experiences regarding the frequency with which participants: *unable to concentrate on their work, lost much sleep over worry, felt they were not playing useful part in things, felt they were unable to make decisions about things, felt unable to overcome difficulties, felt constantly under strain, felt they could not enjoy normal daily activities, felt unable to face their problems, felt unhappy or depressed, lost self confidence, though of themselves as worthless, felt reasonable happy*. By assigning numerical values to the responses, the responses were summed up with higher values indicative of probable cases of emotional distress. As designed and standardized by Goldberg, a cut point of 4 or more on the scale indicates a probable case of emotional distress.^36,37^ The GHQ-12 scale had a Cronbach’s alpha value of 0.84 in Kenya and 0.82 in Uganda.

All three outcome variables (wellbeing (1=Improved wellbeing, 0=No change/Deteriorating wellbeing), self-rated health (1=Good/Very good/Excellent, 0=Fair/Poor) and emotional distress (1=Above cutpoint (≥4), 0=Below cutpoint (<4)) were dichotomous. The independent variables were of either nominal, ordinal, binary interval, or ratio scales. Nominal scale variables included marital status (Partnered, Unpartnered); educational attainment (Some schooling (primary and below), Secondary and above); house ownership (Self-owned, rental). Variables such as household headship, presence of a child/children in the household, presence of persons with disability in the household, whether the participant receives social protection/grant, whether participants spent money to acquire water, and whether the participant had health insurance cover were measured as binary (Yes/No). In terms of empowerment variables, whether participants were responsible for making decisions related to water collection, household water use, the purchase of large household items, running small businesses, and water expenditures was also assessed as binary (Yes/No). Interval-scale variables included the wealth scale, the water and WaSH insecurity scales, while the number of children in the household was assessed as a ratio scale. Moreover, perceived quality and accessibility of healthcare services were assessed using Likert scales ranging from poor to excellent, but dichotomized during analysis into either poor/fair or good/very good/excellent.

### 2.5. Statistical analysis

Descriptive analysis was performed and presented as means, standard deviations, medians and ranges for continuous/interval-scale variables, and frequencies and percentages for categorical variables. Univariable and multivariable regression analysis were performed using a generalized linear model with a complementary loglog link function (binary logistic regression), and the results were presented as Odds Ratios, 95% Confidence Interval and p-values. In developing the multivariable models, variables for inclusion were informed by the feminist political ecology of health framework. Models were assessed to ensure that the residuals were normally distributed, there was no multicollinearity (Variance Inflation Factor, VIF>5), and there were no significant outliers or influential observations. Variables that prevented the model from converging were removed. Furthermore, continuous independent variables, such as wealth and the WASH insecurity index, were linearly correlated with the logit of the outcome variables. Moreover, a mixed-effects logistic regression with ‘country’ as the random effect variable was conducted to investigate the factors associated with improved wellbeing post-COVID-19 pandemic compared to during the pandemic, perceived health relative to others’ own age, and emotional distress, when both the Kenyan and Ugandan data were merged. As the sample sizes could be a potential source of bias, to ensure consistency, the number of predictors in the multivariable models was reduced and results from the fixed effects to the mixed effects logistic regression models were compared. Goodness of fit was assessed using McFadden’s r^2^, marginal r^2^, area under the curve and percentage correctly classified. Excellent models are defined as those with McFadden’s R^2^ between 0.2 and 0.4 or a percentage correctly classified of at least 70%. P-values below 0.05 were indicative of statistical significance, while those between 0.05 and 0.10 were indicative of marginal significance. All analyses were performed using R Software for Statistical Computing (Vienna, Austria), Version 4.4.2.

### 2.6. Ethics

Ethics approval for the study was obtained from University of Waterloo Research Ethics Boards and from the appropriate boards in Uganda and Kenya. All participants provided informed consent prior to participating in the study.

The consent form was integrated into the KoBo collect as the first section of the survey. The survey administrators read the consent to the participants in their local language, and they were given the option to check a box to indicate their agreement to participate.

## 3. Results

The results section begins with an overview of key contextual factors, including sociodemographic characteristics, WASH access and security, and health and wellbeing related variables. The three primary outcomes, self-reported health status, emotional distress and wellbeing, in the context of the COVID-19 pandemic and other variables (i.e. WASH insecurity) are explored. Finally, the results from the multivariable analysis are presented, which investigated how various intersecting factors interacted to further affect the health and wellbeing of older women.

### 3.1. Key contextual factors

#### 3.1.1. Sociodemographic characteristics

Describes the participants’ sociodemographic characteristics. There were 442 female participants aged 60 years and above (231 in Kenya, and 211 in Uganda). 50.7% participants in Uganda were aged between 60 and 70 years, with the proportion being slightly higher in Kenya (53.7%). However, in terms of mean age, the Ugandan participants were relatively older (72.9 years) than the Kenyan participants (69.8 years). The level of education was low, with only 18.2% of the Kenyan sample having secondary or higher education; the level in Uganda was even lower, at 7.1%. There were significant differences between Kenyans and Ugandans across several characteristics. Compared to Ugandan older women, more Kenyan older women were partnered, did not own the houses they lived in, and were not household heads (marginal difference). Moreover, most households in Uganda had children (73%), and the number of children was significantly higher than in Kenya; with 41.6% and 51.2% of the women in Kenya and Uganda, respectively reporting being responsible for looking after children. A significantly higher proportion of the households in Uganda had people living with disability. Approximately a third of the participants in both countries received a social protection grant or food during the COVID-19 pandemic. A higher proportion of the Kenyan women reported a family member, friend or household member had died of COVID-19. 41.6% and 51.2% of the women in Kenya and Uganda, respectively, reported being responsible for looking after children in their households Table 1.

**Table 1.** Sample characteristics (*n* = 442).

​	Kenya	Uganda	*p*-value
	(*N* = 231)	(*N* = 211)	
**Age-group (years)**			
60-70	124 (53.7%)	107 (50.7%)	**0.003**
Above 70	107 (46.3%)	104 (49.3%)	
**Age (years)**			
Mean (SD)	69.8 (6.90)	72.9 (9.64)	0.597
Median [Min, Max]	70.0 [60.0, 96.0]	70.0 [60.0, 105]	
**Highest education level**			
Some Schooling	189 (81.8%)	196 (92.9%)	**0.001**
Secondary and above	42 (18.2%)	15 (7.1%)	
**Marital status**			
Partnered	69 (29.9%)	28 (13.3%)	**<0.001**
Unpartnered	162 (70.1%)	183 (86.7%)	
**House ownership**			
Own by self/family mbr	192 (83.1%)	201 (95.3%)	**<0.001**
Rent/Other	39 (16.9%)	10 (4.7%)	
**Household head**			
No	76 (32.9%)	53 (25.1%)	0.09
Yes	155 (67.1%)	158 (74.9%)	
**Number of children in the household**			
Mean (SD)	2.16 (1.34)	2.92 (2.15)	**0.008**
Median [Min, Max]	2.00 [1.00, 8.00]	2.00 [1.00, 13.0]	
**Children present in the household**			
No	102 (44.2%)	57 (27.0%)	**<0.001**
Yes	129 (55.8%)	154 (73.0%)	
**Household includes people with disability**			
No/Don’t know/No answer	196 (84.8%)	157 (74.4%)	**0.009**
Yes	35 (15.2%)	54 (25.6%)	
**Receives social protection grant/food**			
No	158 (68.4%)	146 (69.2%)	0.938
Yes	73 (31.6%)	65 (30.8%)	
**Responsible for looking after children**			
No	135 (58.4%)	103 (48.8%)	0.053
Yes	96 (41.6%)	108 (51.2%)	
**Someone close died of COVID-19**			
No	182 (78.8%)	189 (89.6%)	**0.003**
Yes	49 (21.2%)	22 (10.4%)	

Bold is used to emphasize the significant values.

#### 3.1.2. WASH access, security and empowerment

Older women in Kenya reported significantly higher levels of group membership than those in Uganda. While the wealth did not differ significantly between the two groups, older women in Kenya had greater access to capital. Decision-making autonomy also varied: a significantly higher proportion of older Kenyan women reported making decisions about water collection, household water use, and small businesses. Notably, more older Ugandan women reported being involved in decisions about large household purchases. Kenyan older women also experienced higher WASH insecurity and water insecurity. Lastly, a significantly greater proportion of Kenyan older women reported spending money on water compared to those living in Uganda (see Table 2).

**Table 2.** WASH access, security and empowerment (*n* = 442).

​	Kenya	Uganda	*p*-value
	(*N* = 231)	(*N* = 211)	
**Wealth index**			
Mean (SD)	4.95 (2.94)	4.45 (2.86)	0.156
Median [Min, Max]	4.00 [0, 12.0]	4.00 [0, 10.0]	
**Makes decision regarding water collection**	n = 69	n = 28	​
No	25 (36.2%)	26 (92.9%)	**<0.001**
Yes	44 (63.8%)	2 (7.1%)	
**Makes decision regarding household water use**	n = 69	n = 28	​
No	12 (17.4%)	4 (14.3%)	**<0.001**
Yes	57 (82.6%)	24 (85.7%)	
**Makes decision regarding purchase of large household items**	n = 69	n = 28	​
No	49 (71.0%)	15 (53.6%)	**0.006**
Yes	20 (29.0%)	13 (46.4%)	
**Makes decision regarding running small businesses**	n = 69	n = 28	​
No	42 (60.9%)	21 (75.0%)	**<0.001**
Yes	27 (39.1%)	7 (25.0%)	
**Makes decision regarding water expenditures**	n = 69	n = 28	​
No	38 (55.1%)	14 (50.0%)	0.096
Yes	31 (44.9%)	14 (50.0%)	
**WASH Insecurity index (High = Less secure)**			
Mean (SD)	11.8 (9.90)	6.64 (8.92)	**<0.001**
Median [Min, Max]	10.0 [0, 33.0]	3.00 [0, 33.0]	
**Water Insecurity index (High = Less secure)**			
Mean (SD)	8.03 (6.64)	5.03 (6.64)	**<0.001**
Median [Min, Max]	8.00 [0, 21.0]	1.00 [0, 21.0]	
**Spend money on water**			
No	42 (18.2%)	164 (77.7%)	**<0.001**
Yes	189 (81.8%)	47 (22.3%)	

*Note. p*-values arise from comparison of sample characteristics between Kenya and Uganda. Bold is used to emphasize the significant values.

#### 3.1.3. Health and wellbeing related variables

Perceptions of health care quality and accessibility were significantly more favourable among Kenyan older women compared to older women in Uganda. Over half of the Kenyan older women in the sample (54.1%) rated the quality of health care services as good, very good, or excellent, compared to only 31.8% in Uganda. Similarly, 68.4% of Kenyans perceived health care services as accessible, versus 27.5% of Ugandan older women. Health insurance coverage was markedly higher in Kenya, with 32.9% insured compared to only 2.8% in Uganda. There was no significant difference in perceived improvement in wellbeing since COVID-19. However, a higher proportion of Ugandan respondents rated their health as fair or poor (75.4%) than Kenyans (52.8%). Emotional distress levels were not significantly different across both countries, with approximately 64% of participants in each group scoring above the distress cutpoint (see Table 3).

**Table 3.** Health and wellbeing related variables (*n* = 442).

​	Kenya	Uganda	*p*-value
	(*N* = 231)	(*N* = 211)	
**Perceived quality of health care services**			
Good/Very good/Excellent	125 (54.1%)	67 (31.8%)	**<0.001**
Poor/Fair	106 (45.9%)	144 (68.2%)	
**Perceived accessibility of health care services**			
Good/Very good/Excellent	158 (68.4%)	58 (27.5%)	**<0.001**
Poor/Fair	73 (31.6%)	153 (72.5%)	
**Has health insurance**			
No	155 (67.1%)	205 (97.2%)	**<0.001**
Yes	76 (32.9%)	6 (2.8%)	
**State of wellbeing has improved since COVID**			
No	190 (82.3%)	180 (85.3%)	0.459
Yes	41 (17.7%)	31 (14.7%)	
**Self-reported health status**			
Fair/Poor	122 (52.8%)	159 (75.4%)	**<0.001**
Good/Very Good/Excellent	109 (47.2%)	52 (24.6%)	
**Emotional distress**			
Below cutpoint (<4)	84 (36.4%)	76 (36.0%)	0.999
Above cutpoint (≥4)	147 (63.6%)	135 (64.0%)	

Bold is used to emphasize the significant values.

### 3.2. Univariable analysis

#### 3.2.1. Self-reported health status

In Kenya, self-reported health status of good/very good/excellent was significantly associated with home ownership and having health insurance. Conversely, having more children, and living with someone with a disability, spending money to acquire water and having had someone close die from COVID were associated with lower odds of reporting better health. In Uganda, the self-reported health status of good/very good/excellent was significantly associated with receiving social protection and having health insurance. Poor perceptions of healthcare quality and accessibility were strongly associated with lower odds of better health in both countries (see Tables 4 and 5).

**Table 4.** Relationships between key contextual factors and outcomes of interest in Kenya (using univariable logistic regression results).

​	Crude odds ratio (OR) (95% confidence interval)		
	Improved wellbeing	Self-reported health status as good/very good/excellent	Emotional distress (≥4)
**Age (yrs)**	0.98 (0.93 - 1.03)	0.56 (0.92 - 1.00) *	1.09 (1.04 -1.14) ***
**Highest education level (Ref: Some)**			
Secondary +	1.88 (0.83 - 4.07)	1.02 (0.52 - 2.00)	1.03 (0.52 - 2.12)
**Marital status (Ref: Partnered)**			
Unpartnered	1.20 (0.56 - 2.55)	0.75 (0.43 - 1.32)	1.68 (0.94 - 2.99).
**House ownership (Ref:Rent/Other)**			
Own	0.80 (0.34 - 1.91)	2.65 (1.28 - 5.85) *	0.11 (0.03 - 0.33) ***
**Household head (Ref:No)**			
Yes	0.72 (0.36-1.47)	1.16 (0.67 – 2.02)	1.22 (0.69 – 2.15)
**Children present in the household (Ref:No)**			
Yes	1.66 (0.83-3.45)	1.44 (0.85 – 2.43)	0.68 (0.39 – 1.17)
**Number of children in the household**	1.08 (0.78 - 1.46)	0.69 (0.51 - 0.92) *	1.36 (1.02 - 1.87) *
**Wealth index**	1.18 (1.05 - 1.32) **	1.08 (0.99 - 1.18)	0.97 (0.89 - 1.06)
**Receives social protection grant/food (Ref:No)**			
Yes	0.88 (0.42 - 1.83)	1.13 (0.65 - 1.98)	1.99 (1.09 - 3.73) *
**Perceived quality of health care services (Ref: Good/Very good/Excellent)**			
Poor/Fair	0.71 (0.35 – 1.41)	0.14 (0.08 – 0.25) ***	3.44 (1.95 – 6.23) ***
**Perceived accessibility of health care services (Ref: Good/Very good/Excellent)**			
Poor/Fair	0.76 (0.34 – 1.57)	0.18 (0.09 – 0.33) ***	3.00 (1.60 – 5.90) ***
**Has health insurance (Ref: No)**			
Yes	2.58 (1.29 – 5.16) **	2.43 (1.39 – 4.31) **	0.30 (0.17 – 0.53) ***
**Household includes people with disability (Ref:No)**			
Yes	0.95 (0.34 – 2.33)	0.19 (0.07 – 0.44) ***	4.05 (1.63 – 12.28) **
**WASH Insecurity index (High = Less secure)**	0.98 (0.94 – 1.02)	0.98 (0.95 – 1.01)	1.13 (1.09 – 1.18) ***
**Water Insecurity index (High = Less secure)**	0.97 (0.92 – 1.03)	0.97 (0.94 – 1.01)	1.17 (1.11 – 1.24) ***
**Spend money on water (Ref:No)**			
Yes	1.36 (0.57 – 3.82)	0.18 (0.08 – 0.39) ***	7.23 (3.48 – 16.03) ***
**Responsible for looking after children (Ref: No)**			
Yes	1.00 (0.50-1.97)	1.21 (0.72 – 2.05)	1.72 (0.99 – 3.01)
**Someone close died of COVID-19 (Ref: No)**			
Yes	1.71 (0.80-3.67)	0.46 (0.24 – 0.90) *	2.31 (1.11 – 4.82) *

*Note.* OR = Odds ratio; Ref = Reference Categories; *p* ≤ 0.10, *p ≤ 0.05, **p ≤ 0.01, ***p ≤ 0.001; CI = confidence intervals. Each row represents the output of a single regression model fitted to the data, with the column header as the dependent variable and the row header as the explanatory variable.

Bold is used to emphasize the significant values.

**Table 5.** Relationships between key contextual factors and outcomes of interest in Uganda (using univariable logistic regression results).

​	Crude odds ratio (OR) (95% confidence interval)		
	Improved wellbeing	Self-reported health status as good/very good/excellent	Emotional distress (≥4)
**Age (yrs)**	1.02 (0.98 - 1.06)	1.00 (0.97 – 1.03)	1.04 (1.00 -1.07) *
**Highest education level (Ref: Some)**			
Secondary +	0.40 (0.02 – 2.08)	0.45 (0.07 – 1.70)	0.83 (0.29 - 2.58)
**Marital status (Ref: Partnered)**			
Unpartnered	0.76 (0.28 - 2.42)	0.98 (0.41 – 2.62)	1.39 (0.61 – 3.12)
**House ownership (Ref:Rent/Other)**			
Own	1.58 (0.28 – 29.64)	0.75 (0.20 – 3.59)	0.19 (0.01 – 1.02)
**Household head (Ref:No)**			
Yes	0.79 (0.35-1.93)	1.34 (0.65 – 2.95)	1.10 (0.57 – 2.09)
**Children present in the household (Ref:No)**			
Yes	1.08 (0.47-2.71)	1.52 (0.74 – 3.33)	0.48 (0.24 – 0.94) *
**Number of children in the household**	1.25 (1.04 - 1.50) *	1.03 (0.87 – 1.21)	1.00 (0.86 - 1.17)
**Wealth index**	0.91 (0.79 - 1.05)	1.05 (0.94 - 1.17)	0.80 (0.72 – 0.89) ***
**Receives social protection grant/food (Ref:No)**			
Yes	1.78 (0.80 – 3.87)	1.98 (1.03 – 3.80) *	0.71 (0.39 – 1.30)
**Perceived quality of health care services (Ref: Good/Very good/Excellent)**			
Poor/Fair	0.70 (0.32 – 1.57)	0.28 (0.14 – 0.53) ***	3.32 (1.82 – 6.13) ***
**Perceived accessibility of health care services (Ref: Good/Very good/Excellent)**			
Poor/Fair	0.64 (0.29 – 1.48)	0.32 (0.16 – 0.62) ***	2.04 (1.10 – 3.80) *
**Has health insurance (Ref: No)**			
Yes	N/A	6.54 (1.24 – 48.24) *	0.27 (0.04 – 1.42)
**Household includes people with disability (Ref:No)**			
Yes	1.75 (0.76 – 3.89)	0.72 (0.33 – 1.50)	0.56 (0.30 -1.05).
**WASH Insecurity index (High = Less secure)**	1.03 (0.99 – 1.07).	0.95 (0.91 – 0.99) *	1.06 (1.03 – 1.11) **
**Water Insecurity index (High = Less secure)**	1.03 (0.97 – 1.08)	0.93 (0.87 – 0.98) *	1.08 (1.03 – 1.14) **
**Spend money on water (Ref:No)**			
Yes	0.47 (0.13 - 1.29)	0.67 (0.28 – 1.44)	0.99 (0.51 – 1.98)
**Responsible for looking after children (Ref: No)**			
Yes	1.02 (0.48 – 2.19)	1.28 (0.68 – 2.40)	0.84 (0.48 – 1.48)
**Someone close died of COVID-19 (Ref: No)**			
Yes	0.55 (0.12 – 2.49)	1.17 (0.43 – 3.15)	0.43 (0.17 – 1.04).

*Note.* OR = Odds ratio; Ref = Reference Categories; *p* ≤ 0.10, *p ≤ 0.05, **p ≤ 0.01, ***p ≤ 0.001; CI = confidence intervals, N/A = Inestimable parameter due to failed convergence Each row represents the output of a single regression model fitted to the data, with the column header as the dependent variable and the row header as the explanatory variable.

Bold is used to emphasize the significant values.

#### 3.2.2. Emotional distress

Probable cases of emotional distress were relatively high in both countries (>60%), with no significant difference between the two countries (see Table 3). In both Kenya and Uganda, emotional distress (score ≥4) was significantly associated with higher WASH and water insecurity. In Kenya, emotional distress was associated with being older, living in rental houses, lack of health insurance, and having had a close person die from COVID (see Table 4). In Uganda, emotional distress was significantly associated with lower wealth, reduced access to capital, and living with children (see Table 5). Poor perceptions of healthcare quality and accessibility were strong predictors of distress in both countries. These results underscore the multifaceted nature of emotional distress, influenced by economic, demographic, and psychosocial factors.

#### 3.2.3. Wellbeing

In Kenya, improved wellbeing was significantly associated with more wealth, greater access to capital, and having health insurance. Additionally, in Kenya, greater leisure time was marginally associated with improved wellbeing (See Table 4). By comparison, in Uganda, improved wellbeing was positively associated with the number of children in the household (see Table 5).

### 3.3. Multivariable analysis

Table 6 shows the multivariable regression results for factors associated with an improvement in wellbeing post-pandemic compared with during the pandemic, as well as self-reported health status and emotional distress, after adjusting for other potential confounding factors. In Kenya, post-pandemic improvements in wellbeing were associated with wealth and health insurance. Self-reported health status as good/very Good/excellent was associated with perceived good quality of healthcare services, not living with persons with disability and not having to spend money to acquire water. Emotional distress was reportedly higher among older women, those who lived in rental houses rather than in their own houses, those who were receiving social protection grants/food, those who reported water insecurity, and those who had to spend money to acquire water. In Uganda, on the other hand, post-pandemic improvements in wellbeing were not associated with any of the variables, whereas better health was associated with perceived good/very good/excellent quality of healthcare services. Emotional distress was associated with less wealth, perceived poor quality of healthcare services, and reporting water insecurity (see Table 6).

**Table 6.** Factors associated with wellbeing improvement before to after COVID-19, Perceived health relative to others of same age, and emotional distress in Kenya and Uganda (using multivariable logistic regression results).

​	Improved wellbeing		Self-reported health status (good/very good/excellent)		Emotional distress (≥4)	
	Adjusted OR (95% CI)					
	Kenya	Uganda	Kenya	Uganda	Kenya	Uganda
**Age (yrs)**	1.01 (0.96-1.07)	1.00 (0.95-1.05)	0.99 (0.94-1.04)	0.99 (0.95-1.04)	1.12(1.05-1.21) **	1.04 (1.00-1.09).
**Highest education level (Ref: Some)**						
Secondary +	1.37 (0.52-3.40)	0.62 (0.03-4.07)	1.45 (0.59-3.60)	0.37 (0.05-1.72)	1.16 (0.41-3.34)	1.88 (0.50-7.47)
**Marital status (Ref: Partnered)**						
Unpartnered	3.26 (0.97-11.5)	0.58 (0.14-2.54)	0.37 (0.10-1.21)	0.82 (0.21-3.28)	1.38 (0.37-5.78)	0.78 (0.22-2.64)
**House ownership (Ref:Rent/Other)**						
Own	1.00 (0.37-2.89)	1.45 (0.22-29.8)	1.47 (0.58-3.81)	0.79 (0.18-4.26)	0.12(0.02-0.42) **	0.21 (0.01-1.37)
**Household head (Ref: No)**						
Yes	0.23 (0.07-0.73) *	0.88 (0.30-2.82)	2.40 (0.75-8.83)	1.18 (0.41-3.72)	1.14 (0.27-4.35)	1.24 (0.45-3.34)
**Children present in the household (Ref:No)**						
Yes	1.64 (0.65-4.22)	1.33 (0.43-4.30)	1.65 (0.72-3.88)	1.50 (0.58-4.01)	0.42 (0.15-1.18)	0.46 (0.18-1.13)
**Wealth index**	1.18 (1.03-1.37) *	0.94 (0.80-1.11)	1.12 (0.99-1.27).	1.01 (0.88-1.15)	0.95 (0.82-1.09)	0.83 (0.72-0.94) **
**Receives social protection grant/food (Ref:No)**						
Yes	0.83 (0.36-1.83)	1.48 (0.57-3.76)	1.02 (0.50-2.07)	1.77 (0.78-4.02)	2.70(1.15-6.64) *	0.46 (0.19-1.11).
**Perceived quality of health care services (Ref: Good/Very good/Excellent)**						
Poor/Fair	0.61 (0.27-1.34)	0.82 (0.32-2.13)	0.13(0.06-0.26) ***	0.31 (0.14-0.67) **	2.63 (1.19-5.97) *	2.91(1.35-6.43) **
**Has health insurance (Ref: No)**						
Yes	2.29(1.00-5.36) *	N/A	1.61 (0.72-3.60)	N/A	0.94 (0.40-2.22)	N/A
**Household includes people with disability (Ref:No)**						
Yes	0.75 (0.24-2.03)	1.74 (0.70-4.22)	0.15(0.05-0.44) ***	0.45 (0.18-1.05).	2.98 (0.95-10.89).	0.63 (0.29-1.34)
**WASH Insecurity index (High = Less secure)**	1.00 (0.95-1.04)	1.04 (0.99-1.08).	1.01 (0.97-1.04)	0.96 (0.91-1.00).	1.12 (1.07-1.19) ***	1.06 (1.01-1.12) *
**Spend money on water (Ref:No)**						
**Yes**	1.75 (0.60-5.83)	0.51 (0.14-1.50)	0.35(0.12-0.95) *	0.92 (0.35-2.22)	5.35(1.86-16.58) **	0.80 (0.35-1.87)
**Responsible for looking after children (Ref: No)**						
Yes	0.77 (0.30-1.99)	0.97 (0.36-2.72)	1.18 (0.50-2.80)	1.20 (0.52-2.82)	1.82 (0.60-5.53)	1.61 (0.70-3.76)
**Someone close died of COVID-19 (Ref: No)**						
Yes	1.13 (0.44-2.78)	0.44 (0.06-1.99)	0.45 (0.18-1.08).	1.85 (0.54-5.90)	1.17 (0.42-3.31)	0.46 (0.14-1.40)
**Model Statistics**						
Mc Fadden R^2^	0.110	0.067	0.290	0.113	0.412	0.193
Area Under Curve	0.728	0.661	0.838	0.718	0.898	0.622
Classification	0.741	0.765	0.674	0.679	0.674	0.645

*Note.* OR = Odds ratio; Ref = Reference Categories; *p* ≤ 0.10, *p ≤ 0.05, **p ≤ 0.01, ***p ≤ 0.001; CI=confidence intervals. Each row represents the output of a single regression model fitted to the data, with the column header as the dependent variable and the row header as the explanatory variable. N/A = Not-estimable parameters due to failed convergence. Goodness of fit measures: Mc Fadden R2, Area under curve, and Percentage correctly classified.

Bold is used to emphasize the significant values.

Table 7 presents the results of the mixed-effects model after combining the two datasets to address potential sample inadequacy. In the multivariable models, improved wellbeing post-pandemic, when compared to during the pandemic, was significantly associated with having health insurance. Better health relative to others’ own age was associated with perceiving the quality of healthcare services as good/very good/excellent, having health insurance, and not having persons with disability within the household. Moreover, emotional distress was associated with older women, living in rental houses, poor/fair perceived quality of healthcare services, and WASH insecurity (see Table 7).

**Table 7.** Factors associated with wellbeing improvement before to after COVID-19, perceived health relative to others of same age, and emotional distress in Kenya and Uganda (using univariable and multivariable mixed effects logistic regression results).

​	Improved wellbeing		Self-reported health status (good/very good/excellent)		Emotional distress (≥4)	
	Adjusted OR (95% CI)					
	Univariable	Multivariable	Univariable	Multivariable	Univariable	Multivariable
**Age (yrs)1**	1.00 (0.98-1.03)	1.01 (0.98-1.05)	0.98 (0.96-1.00)	0.99 (0.97-1.02)	1.03(1.02-1.05) ***	1.03(1.01-1.06) ***
**Highest education level (Ref: Some)1**						
Secondary +	1.40 (0.75-2.60)	1.31 (0.65-2.65)	0.93 (0.58-1.47)	0.93 (0.55-1.57)	0.98 (0.68-1.41)	1.09 (0.69-1.72)
**Marital Status (Ref: Partnered)1**						
Unpartnered	0.98 (0.56-1.71)	1.48 (0.68-3.23)	0.83 (0.58-1.20)	0.56 (0.29-1.08).	1.33 (0.98-1.81).	1.15 (0.68-1.95)
**House ownership (Ref:Rent/Other)1**						
Own	0.86 (0.43-1.73)	0.85 (0.40-1.78)	1.80(1.03-3.16) *	1.33 (0.73-2.43)	0.37(0.25-0.55) ***	0.32(0.19-0.54) ***
**Children present in the household (Ref:No)1**						
Yes	1.31 (0.79-2.16)	1.39 (0.78-2.50)	1.33 (0.95-1.86).	1.29 (0.84-1.98).	0.73(0.57-0.94) *	0.66(0.46-0.95)*
**Household head (Ref: No)**						
Yes	0.76 (0.46-1.23)	0.55 (0.29-1.07).	1.16 (0.82-1.64)	1.52 (0.82-2.81)	1.10 (0.84-1.45)	1.02 (0.64-1.63)
**Wealth index**	1.06 (0.98-1.14)	1.06 (0.97-1.16)	1.06 (1.00-1.11)	1.03 (0.97-1.10)	0.93(0.89-0.97) ***	0.95 (0.89-1.00).
**Receives social protection grant/food (Ref:No)1**						
Yes	1.19 (0.73-1.93)	1.07 (0.65-1.78)	1.30 (0.94-1.80)	1.11 (0.77-1.59)	1.12 (0.86-1.45)	1.07 (0.77-1.48)
**Perceived quality of health care services (Ref: Good/Very good/Excellent)1**						
Poor/Fair	0.70 (0.44-1.12)	0.74 (0.45-1.22)	0.26(0.18-0.37) ***	0.25(0.17-0.37) ***	2.07(1.60-2.68) ***	2.07(1.52-2.82) ***
**Has health insurance (Ref: No)1**						
Yes	1.94(1.16-3.22) *	1.81(1.02-3.23) *	2.14(1.46-3.14) ***	1.94 (1.18-3.18) **	0.49(0.33-0.73) ***	0.76 (0.50-1.14)
**Household includes people with disability (Ref:No)1**						
Yes	1.25 (0.73-2.16)	1.23 (0.70-2.17)	0.43(0.26-0.72) ***	0.39(0.23-0.66) ***	1.09 (0.81-1.47)	1.03 (0.73-1.46)
**WASH Insecurity index (High = Less secure)1**	1.01 (0.98-1.03)	1.02 (0.99-1.04)	0.98(0.96-1.00) *	1.00 (0.98-1.02)	1.05(1.03-1.06) ***	1.04 (1.03-1.06) ***
**Spend money on water (Ref:No)1**						
**Yes**	1.03 (0.65-1.65)	0.91 (0.54-1.55)	0.41(0.28-0.62) ***	0.77 (0.45-1.32)	1.55(1.12-2.15) **	**1.33(0.97-1.83).**
**Responsible for looking after children (Ref: No)**						
Yes	0.99 (0.62–1.57)	0.91 (0.52-1.59)	1.17 (0.86-1.61)	1.21 (0.79-1.83)	1.13 (0.88-1.44)	1.38 (0.95-1.99).
**Someone close died of COVID-19 (Ref: No)**						
Yes	1.29 (0.72-2.32)	1.03 (0.54-1.96)	0.66 (0.42-1.05).	0.68 (0.41-1.12)	1.13 (0.82-1.57)	0.99 (0.65-1.50)
**Model Statistics**						
Mc Fadden R^2^	​	0.040	​	0.198	​	0.239
Marginal R^2^	​	0.114	​	0.354	​	0.332

*Note.* OR = Odds ratio; Ref = Reference Categories; *p* ≤ 0.10, *p ≤ 0.05, **p ≤ 0.01, ***p ≤ 0.001; CI=confidence intervals. Each row represents the output of a single regression model fitted to the data, with the column header as the dependent variable and the row header as the explanatory variable. Goodness of fit measures: Mc Fadden R2, Area under curve, and Percentage correctly classified.

Bold is used to emphasize the significant values.

## 4. Discussion

This research presented findings from a quantitative investigation examining how socioeconomic factors impact health and wellbeing outcomes among older women living in Kenya and Uganda post COVID-19. There are several significant factors impacting older women’s self-reported health status, emotional distress and wellbeing.

**Figure 2.**
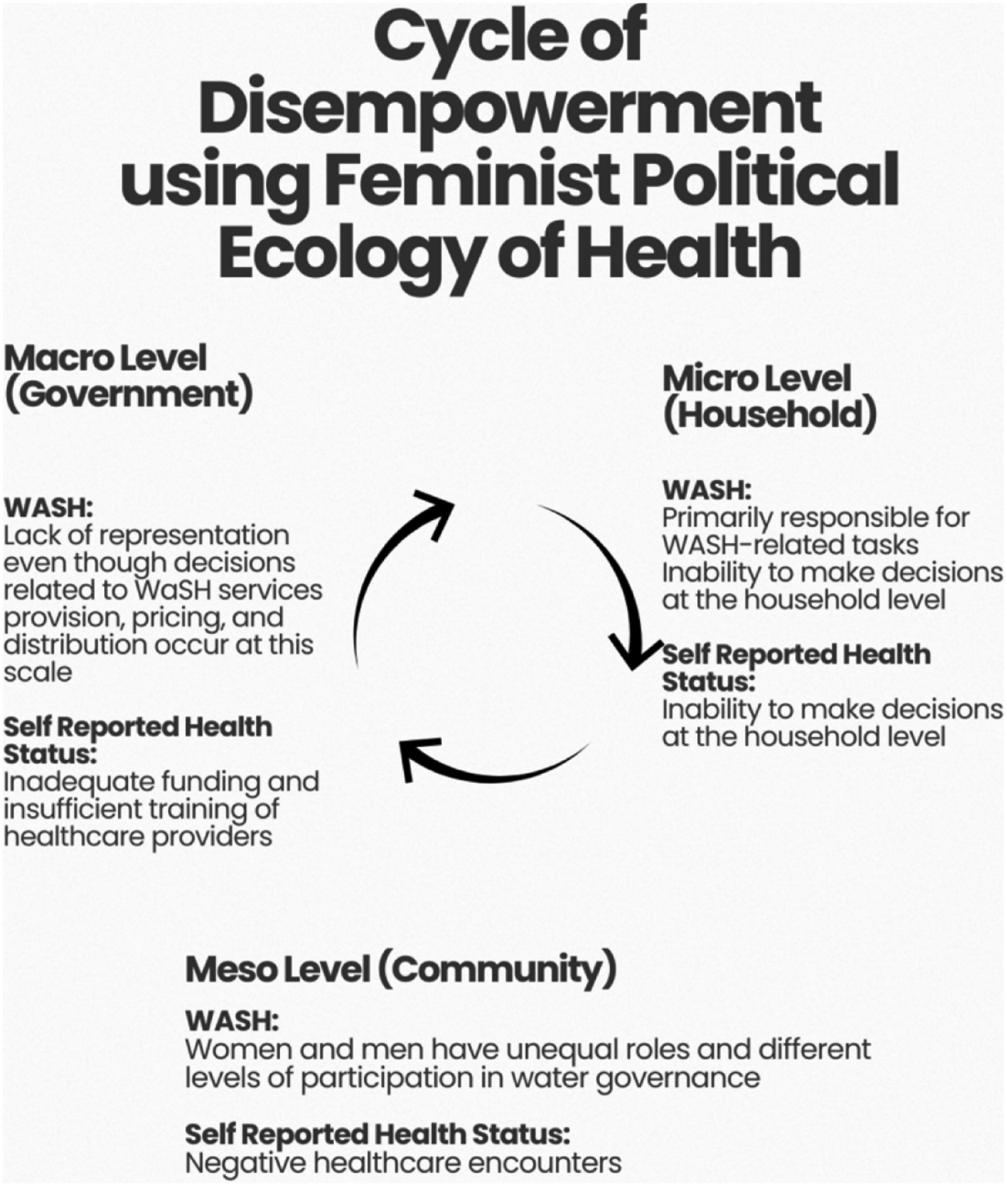
Cycle of disempowerment using feminist political ecology of health.

The majority of older women in both Uganda (75%) and Kenya (53%) reported that their health status was fair/poor compared with others of the same age. In comparison, at the height of the pandemic, 60% of older adults in Uganda perceived their health as fair/poor.^20^ Another study focusing on 18 countries in Africa, including Kenya and Uganda, stated that 81.1% of individuals reported their general health as ‘good’ or ‘very good’ during the early phase of the COVID-19 pandemic.^38^ The high levels of fair/poor self-perception of health status among older women in Uganda and Kenya can be attributed to several variables, as highlighted in the research (see Figure 2). For instance, in both countries, the perception of good quality healthcare services was associated with better relative perceived health. The feminist political ecology of health framework highlights how multiple scales (i.e., micro, meso, and macro) are interconnected and produce feedback that reinforces vulnerabilities and perpetuates the cycle of disempowerment.^24^ For example, at the macro level, inadequate funding and insufficient training of healthcare providers in addressing the specific needs of older women often result in substandard care.^8^ At the meso level, this causes negative healthcare encounters in the community, such as being dismissed, misunderstood, or not taken seriously, which reinforces stigma, decreases trust, discourages care-seeking behaviors, and contributes to negative health outcomes among this population.^39^ To further highlight, research has shown that when older women believe healthcare services are of good quality, they have greater trust in healthcare providers, leading to improved adherence to medical recommendations.^40^ Older women may be more likely to seek preventive care if they have access to high-quality care, including feeling respected and heard in the healthcare setting.^41^ Based on these findings, there should be a focus on centring the needs of older women by addressing their feelings and fears and building trust within the healthcare systems.^42^ Thus, it is recommended that healthcare providers and community health workers receive geriatric healthcare training to better understand the needs of older women.^8^

The analysis also underscored that, in both Uganda and Kenya, older women who were able to make decisions about purchasing large household items had better self-reported health status. Studies situated in SSA have highlighted how engaging in decision-making at the household level improves several health outcomes.^43,44^ Having a say in one’s household helps foster a sense of autonomy and self-worth, which are key factors in overall health.^43,44^ Thus, to improve women’s engagement in decision-making, interventions and policies should focus on improving household gender relationships, empowering women, and shifting social norms.^45^

Furthermore, the analysis found that 60% of older women in both countries continue to experience high levels of emotional distress even after the height of the COVID-19 pandemic. Previous research conducted in Uganda during the height of the pandemic indicated that 87% of individuals aged 60 years or older had emotional distress.^20^ In Kenya, 21.7% of women sampled during the COVID-19 pandemic had emotional distress.^46^ Comparatively, in Ghana, before the COVID-19 pandemic, it was found that 35% of individuals had emotional distress.^47^ Further, other studies have underscored that, although there were challenges before the COVID-19 pandemic, the pandemic truly amplified pre-existing structural inequities.^15,48^ For example, due to the increase in domestic work during the COVID-19 pandemic, there was also an increase in depressive and anxiety symptoms among women living in Africa.^49^ The persistent, heightened levels of emotional distress and a lack of improvement in wellbeing levels since the pandemic among older women in Uganda and Kenya are in part due to the COVID-19 pandemic and its associated public health measures, which exposed and amplified the vulnerabilities that older adults in SSA face.^15^ Using the feminist political ecology of health framework, it can be underscored that the political structures and power relations in place deprioritize women.^24^ Hence, despite the negative impacts of the pandemic, there was limited assistance from governments in terms of social protections and targeted interventions,^50^ which may explain why the effects of the pandemic remain persistent. The findings indicate the need for a larger focus on advocating for increased support from the government and local leaders to support older women’s wellbeing and emotional distress.^51^

Finally, the last objective was to focus on WASH amenities as a determinant of health and wellbeing for older women in Kenya and Uganda. The analysis found that water insecurity was associated with higher odds of emotional distress among older women in Kenya and with poorer health in Uganda. Recent studies across Sub-Saharan Africa^47,52^ have highlighted the linkages between WASH insecurity and emotional distress. The feminist political ecology of health framework highlights that water is a gendered resource and that water insecurity causes women to experience disproportionate stress due to the level of responsibility they bear for WASH-related tasks.^24^ Despite these significant responsibilities, women frequently lack decision-making power at both macro and meso levels^53^ (see Figure 2). Notably, older women in Uganda experienced improvements in well-being when they were able to make decisions about household-level water collection. When women have a voice in decision-making, they experience greater autonomy and self-control.^54^ Interestingly, in Kenya, women who made fewer decisions about water collection reported better health. This difference may be explained by the fact that, in Kenya, individuals are more likely to pay for water than those in Uganda.^55^ The financial burden of purchasing water can increase stress, which, in turn, may negatively impact health. This added stress and sense of insecurity, particularly for women who are often responsible for WASH-related tasks, could contribute to poorer health status.^56^ To generate targeted solutions and empower women, older women’s voices and perspectives must be included in discussions to ensure their WASH needs are met.

### 4.1. Limitations and directions for future research

Several limitations should be noted, which also point to important directions for future research. First, recall bias may have influenced responses, as the surveys were administered after the peak of the COVID-19 pandemic. Older women’s self-reported data may also have been affected by social desirability bias, particularly regarding WASH practices and health perceptions. In addition, the sampling approach may have introduced selection bias and interviewer bias is also possible, given that surveyors may have had prior familiarity with participants.

Due to the cross-sectional design, causal relationships between variables cannot be established. Moreover, the small sample sizes limit the ability to detect within-country effects and restrict the generalizability of the findings to broader populations. To address these limitations, future research would benefit from longitudinal study designs with larger samples. Moreover, our survey did not measure food insecurity, which is closely related to health and wellbeing. Future studies should also measure food insecurity to better understand the relationship between food insecurity, health, wellbeing and emotional distress during a global shock.

There are also temporal limitations, as post–COVID-19 findings may not be applicable to future shocks. As such, future research should explicitly adopt comparative and longitudinal approaches that examine how older women experience and respond to different global shocks over time. Another recommendation would be to employ alternative epistemological approaches (e.g., focus groups and oral histories) to better capture the nuanced experiences of older women living in SSA during the COVID-19 pandemic and other global shocks.

### 4.2. Practical and theoretical significance

Despite the aforementioned limitations, this study makes a unique contribution by documenting how older women in Uganda and Kenya have been and continue to be affected by the COVID-19 pandemic. While prior research has broadly examined the impacts of COVID-19 on older adults, this study centers on older women, a group that is particularly vulnerable during global shocks yet often underrepresented in research. The study demonstrates that the majority of older women in both countries reported fair or poor health, alongside persistently high levels of emotional distress. Notably, the prevalence of fair or poor self-rated health and emotional distress observed in this study exceeds levels reported in much of the existing literature.

Interpreted through the feminist political ecology of health framework, our results reflect the structural conditions that systematically deprioritize older women. For example, the current healthcare systems and insufficient training of healthcare providers contribute to negative healthcare encounters, discouraging older women from seeking care and exacerbating adverse health outcomes. These challenges were further intensified during the COVID-19 pandemic, as fear of infection and the closure of public transportation compounded existing barriers to healthcare access.^15^ In response, this study underscores the importance of strengthening geriatric care training among healthcare providers and community health workers, as well as utilizing community health workers to bring healthcare services closer to older women.^57^

Further, by grounding the analysis in the feminist political ecology of health framework, this study interprets empirical findings through a power- and scale-sensitive lens and extends the framework in various ways. In particular, this research highlights the role of age in producing gendered health inequities. Older women have distinct experiences as a result of both their age and gender. This work also introduces the cycle of disempowerment (Figure 2) to capture how vulnerabilities are reproduced across scales. The framework and the cycle of disempowerment illuminate how gender and health are interconnected at the micro, meso, and macro levels. At the macro scale, for example, older women remain largely excluded from decision-making around WASH services despite bearing primary responsibility for WASH-related labour. This structural disempowerment reinforces negative health and wellbeing outcomes, which are then reproduced across scales and over time.

These insights are crucial for advancing both theory and practice. It is necessary to meaningfully integrate older women’s perspectives into policy and decision-making processes at all levels, particularly regarding WASH and health systems planning.

## 5. Conclusion

The study focused on the impact of the COVID-19 pandemic on the health and wellbeing of older women living in Uganda and Kenya. Through the feminist political ecology of health framework, this research illustrated the several negative impacts of the COVID-19 pandemic on older women’s self-reported health status, emotional distress and wellbeing. To highlight, a high percentage of older women continue to experience emotional distress even after the COVID-19 pandemic is no longer considered a global emergency. Hence, there are several key recommendations, including advocating for increased support from government agencies to implement targeted solutions that mitigate the impacts of the pandemic on older women. Furthermore, it is crucial to prioritize older women’s voices in research and practice to ensure their needs are met. Finally, considering the rapidly aging population in SSA and future shock preparedness, including emerging pandemics and climate-related disasters, increased global attention needs to be paid to vulnerable populations, such as older women.

## Data Availability

The data that support the findings of this study are available on request from the corresponding author, [SD].*
